# Impact of zearalenone on quorum sensing signaling molecules and its association with the suppression of ruminal microbial fermentation in a RUSITEC system

**DOI:** 10.1186/s40104-025-01337-z

**Published:** 2026-01-24

**Authors:** Zuo Wang, Tianyi Ma, Jianhua He, Yu Ge, Qianglin Liu, Xinyi Lan, Lei Liu, Fachun Wan, Weijun Shen

**Affiliations:** 1 Yuelushan Laboratory, College of Animal Science and Technology, Hunan Agricultural University, Changsha, Hunan 410128 People’s Republic of China; 2 College of Veterinary Medicine, Hunan Agricultural University, Changsha, Hunan 410128 People’s Republic of China

**Keywords:** Acyl-homoserine lactones, Autoinducer-2, Quorum sensing, Rumen microbiome, Zearalenone

## Abstract

**Background:**

Zearalenone (ZEN), a common mycotoxin in ruminant diets, could disturb the rumen ecosystem and impair rumen fermentation. Noticeably, ZEN has been shown to reduce the relative abundances of specific bacterial taxa that potentially possess quorum sensing (QS) functions, which are deemed essential for the microbial interactions and adaptations during rumen fermentation. Nonetheless, whether QS communications participate in the responses of rumen microbial fermentation to ZEN remains unknown. Therefore, the present trial was performed to explore the potential roles of QS during the alterations of rumen microbial fermentation by ZEN through a rumen simulation technique (RUSITEC) system, in a replicated 4 × 4 Latin square design.

**Results:**

ZEN significantly (*P* < 0.05) reduced QS signal autoinducer-2 (AI-2), and tended to (*P* = 0.051) downregulate QS signal C4-homoserine lactone (HSL). ZEN also significantly (*P* < 0.05) decreased total volatile fatty acid (TVFA), acetate, propionate, isobutyrate, isovalerate, organic matter disappearance (OMD), neutral detergent fiber disappearance (NDFD), and acid detergent fiber disappearance (ADFD) in different manners. The linear discriminant analysis effect size (LEfSe) analysis indicated significantly (*P* < 0.05) differential enrichments of a series of bacterial taxa such as *Butyrivibrio_sp_X503*, *Rhizobium daejeonense*, *Hoylesella buccalis*, *Ezakiella coagulans*, *Enterococcus cecorum*, *Ruminococcus_sp_zg-924*, *Polystyrenella longa*, and *Methylacidimicrobium fagopyrum* across different treatments. The phylogenetic investigation of communities by reconstruction of unobserved states 2 (PICRUSt2) analysis suggested that QS were predicted to be significantly (*P* < 0.05) affected by ZEN. The metabolomics analysis detected considerable significantly (*P* < 0.05) differing metabolites and implied that ZEN challenge significantly (*P* < 0.05) influenced the indole alkaloid biosynthesis, biosynthesis of alkaloids derived from shikimate pathway, and sesquiterpenoid and triterpenoid biosynthesis. Significant (*P* < 0.05) interconnections of QS molecules with the differential rumen fermentation traits, differential bacterial taxa, and differential metabolites were exhibited by Spearman analysis.

**Conclusions:**

ZEN negatively affected the QS signals of AI-2 and C4-HSL, which was found to correlate with the fluctuations in specific rumen fermentation characteristics, ruminal bacterial populations, and ruminal metabolisms. These interrelationships implied the potential involvement of QS in the reactions of rumen microbiota to ZEN contamination, and probably contributed to the inhibition of rumen fermentation.

**Supplementary Information:**

The online version contains supplementary material available at 10.1186/s40104-025-01337-z.

## Background

Zearalenone (ZEN), a secondary metabolite of the *Fusarium* spp., is a mycotoixn frequently detected in the livestock feedstuffs worldwide [1]. Owing to the estrogenic effects of ZEN and its metabolites, the chronic exposure to this contaminant would lead to reproductive disturbances such as ovarian cyst, hyperestrogenism with false estrus, and vulva swelling of female animals [2]. Ruminants had been previously considered more tolerant to the mycotoxicosis compared to other domestic animals, as the ruminal microorganisms can, to a certain extent, decompose various mycotoxins and thence serve as a biological barrier [3]. Interestingly, it was discovered by Hartinger et al. [4] that the short-term exposure to ZEN decreased the ruminal pH and total short-chain fatty acid concentration, as well as the relative abundances of Lachnospiraceae and Prevotellaceae, suggesting that ZEN could still interrupt the ruminal microbial fermentation despite the degradation of this mycotoxin by the rumen microbes [3]. Nonetheless, how ZEN exerts impacts on the rumen microflora and relevant ruminal metabolisms is currently unclear.

Quorum sensing (QS) is a widespread cell-to-cell communication strategy amongst the diverse microbial populations, and it is mediated by self-synthesized signaling molecules termed as the autoinducers [5, 6]. Via autoinducers, QS can enable various microbes to dynamically adjust their behaviors in response to the ambient cell density [7]. After sufficient quantity of autoinducers are secreted, the successive binding with specific receptors and signal transduction cascade would lead to the regulations of a series of physiological processes, such as the growth, proliferation, motility, biofilm formation, virulence discharge, extracellular protease production, and antibiotic resistance [8–10]. In view of the indispensable significance of rumen microbial consortia in the symbiotical biofilm formation and biomass conversion during feed digestion [11, 12], there are increasing evidences and consensuses that QS which facilitates intricate microbial communications and coordinations might play a crucial role in shaping the rumen fermentation pattern and ruminant productivity [7, 10, 13–16].

In regard to the rumen bacterial microflora, most of the investigations aiming at QS have focused on either the acyl-homoserine lactones (AHLs)-based QS system or the autoinducer-2 (AI-2)-mediated QS pathway [5, 14]. The AHLs-regulated QS is prevailing within the Gram-negative bacteria as an intraspecies communication mechanism, and its signaling molecules have been consecutively identified in bovine rumen by Erickson et al. [17] and Edrington et al. [18]. Subsequently, Yang et al. [6] reported the isolation and characterization of a *Pseudomonas aeruginosa* strain YZ1 from cattle rumen, whose capacity to produce at least three types of AHLs was also verified. Moreover, analysis targeting the AHL genes based on 448 rumen bacterial genomes found that only one Gram-negative species, i.e., *Citrobacter* sp. NLAE-zl-C269, seemed to be capable of synthesizing AHLs [5].

By contrast with the AHLs, AI-2 is commonly generated by both Gram-negative and Gram-positive bacteria and it could serve as a universal agent for interspecies communications of the rumen microbiota [19, 20]. The AI-2-like molecules was firstly detected by Mitsumori et al. [21] from the culture fluids of four ruminal bacterial species (i.e., *Butyrivibrio fibrisolvens*, *Eubacterium ruminantium*, *Ruminococcus flavefaciens*, and *Succinimonas amylolytica*), and the genes (*luxS*) encoding the AI-2 synthase (LuxS) were further mined from the ruminal microbiome through metagenomic and metatranscriptomic approaches [16]. More recently, Won et al. [5] and Liu et al. [10] successively discovered that both the metagenome and metatranscriptome datasets of rumen microbiota were dominated by the AI-2-relavant genes, suggesting that the AI-2-mediated QS could be more prevalent in the rumen microbial social networks than the AHLs-based QS.

As QS systems are essential for the self-adaptation and self-defense of microbes under varying ambiences [22], dietary alterations that significantly affects the ruminal microbial communities would probably trigger the response of QS communication. For instance, influences from the varying ratio of concentrate to forage on the QS signals of AHLs [17] and AI-2 [13] within the rumen microbiome have been reported, respectively. It could be further speculated that diet contamination by the mycotoxins possessing antimicrobial capacity could also arouse the QS mechanisms, since one of the main purposes of QS is to secure the survival of bacterial populations during environmental deterioration [22, 23]. Being an antimicrobial mycotoxin, ZEN has been verified to decrease the microbial diversity [24] and lower the relative abundances of Lachnospiraceae and Prevotellaceae [4] in the rumen microflora. Noticeably, the positive correlation between Lachnospiraceae and AI-2-mediated QS has been observed in precedent investigations [25, 26]. Besides, the genus *Prevotella*, a major subordinate of Prevotellaceae inhabiting the rumen ecosystem, has been identified with highly abundant and expressed *luxS*-related AI-2 QS genes [5, 10]. Nevertheless, whether the QS communications really participates in the detriment of rumen microbial fermentation by ZEN still remains uncertain.

Therefore, the hypothesis that QS could be involved in the reactions of ruminal microbiota to the ZEN contamination was proposed. The present trial was therefore conducted by adopting a well-developed rumen simulation technique (RUSITEC) system that can exclude interventions from irrelevant factors, through which the influences of ZEN challenge on QS signaling molecules, rumen fermentation profiles, rumen microbiome, and relevant metabolisms were investigated.

## Methods

### Animals, diets, and management

The current trial was supervised and approved by the Animal Care Committee (approval number: 20241003), College of Animal Science and Technology, Hunan Agricultural University (Changsha, China). A total of six rumen-fistulated Xiangxi yellow bulls (initial body weight: 296 ± 31.3 kg (mean ± SE), and 46 ± 1 months of age) were used as donors for the rumen contents. Being housed in a tie-stall barn, all the donors were ad libitum fed a basal total mixed ration (TMR; Table 1) twice daily (08:00 h and 20:00 h), and guaranteed free access to fresh water.

**Table 1:** Constituents and chemical composition of the basal TMR substrate

Ingredient, g/kg DM		Chemical composition, g/kg DM	
Maize straw	750.0	OM	855.0
Corn meal	126.2	CP	76.4
Wheat bran	6.2	NDF	599.8
Rice bran	12.3	ADF	304.5
Corn husk	9.9	EE	13.2
Rice bran meal	24.8	Ash	145.0
Soybean meal	16.1	Ca	6.8
Cottonseed meal	7.4	P	3.2
DDGS	24.7		
Molasses	2.5		
CaCO_3_	9.4		
NaHCO_3_	2.5		
NaCl	2.5		
Ca(H_2_PO4)_2_	1.5		
Premix^1^	4.0		

^1^Every 1 kg of premix contained 260 mg of Cu, 3,200 mg of Fe, 160 mg of Mn, 10 mg of I, 3 mg of Co, 3 mg of Se, 20,000 IU of vitamin A, 800 IU of vitamin D_3_, and 75 IU of vitamin E

*OM *Organic matter, *CP *Crude protein, *NDF *Neutral detergent fiber, *ADF *Acid detergent fiber, *EE *Ether extract, *DDGS* Distiller’s dried grains with solubles

### RUSITEC fermentation

Before morning feeding of the first day in each RUSITEC fermentation period, the rumen contents of all the bulls were collected from different sites, i.e., the cranial sac, caudodorsal blind sac, caudoventral blind sac, dorsal sac, and ventral sac of the rumen, and then strained through 4 layers of cheesecloth under a continuous CO_2_ stream. Two liters of rumen fluid from each donor were obtained and evenly mixed, and immediately transported to the laboratory in an anaerobic container prewarmed at 39 °C. Subsequently, the strained rumen liquid was equally mixed with prewarmed McDougall’s buffer [27] to prepare the buffered rumen fluid, and then the RUSITEC fermentation system was constructed and operated as per the procedures and settings established in earlier studies [28, 29]. The current trial was conducted following a replicated 4 × 4 Latin square design, and each vessel was sequentially allocated to each of the 4 treatments through the 4 experimental periods. Each experimental period consisted of a 4-day adaptation phase (d 1–4) followed by a 3-day sampling phase (d 5–7). During this experiment, a sum of 12 fermentation vessels were synchronously used and randomly assigned to 4 groups: the control (the basal substrate, CON), the low-dosage ZEN treatment (the basal substrate supplemented with ZEN at 400 μg/kg dry matter [DM], ZENL), the mid-dosage ZEN treatment (the basal substrate supplemented with ZEN at 800 μg/kg DM, ZENM), and the high-dosage ZEN treatment (the basal substrate supplemented with ZEN at 1,600 μg/kg DM, ZENH), with 3 vessels employed in each treatment. The basal substrate for RUSITEC fermentation was exactly the same as the TMR diet provided for the donor cattle, and the ZEN (purity 99.9%) was purchased from the Alta Scientific Co., Ltd. (Tianjin, China). During the fermentation, 20 g of the basal substrate, with or without the ZEN supplementation, was imported into every vessel twice daily at 08:00 h and 20:00 h.

### Sample collection

The samples of RUSITEC fermentation was collected according to the methods of previous reports [28, 29] with slight modifications. To prepare for the assessments of the ruminal pH, ammonia nitrogen (NH_3_-N), volatile fatty acids (VFAs), microbial protein (MCP), lipopolysaccharide (LPS), and QS signaling molecules, rumen fluid samples from each vessel were collected daily via the overflow port prior to the morning feeding during sampling phase, and then evenly combined. To measure the disappearances of nutrients during fermentation, the rumen solids were collected daily through the discharge outlets throughout the sampling period. As to the preparation for analysis of both full-length 16S rRNA gene sequencing and ruminal metabolomes, 2 vessels from each treatment in each experimental period were randomly selected and their rumen fluids collected daily during the sampling phase were then pooled uniformly. All the above samples were stored at −80 °C until further analysis was carried out.

### Chemical and biochemical analysis

Nutrients in the TMR and rumen solids were analyzed through approaches described by AOAC [30] and precedent studies [31, 32]. The evaluations for ruminal pH, NH_3_-N, VFA, and MCP were performed following the instructions adopted by Wang et al. [29]. A chromogenic endpoint Tachypleus Amebocyte Lysate assay kit (EC80545S, Chinese Horseshoe Crab Reagent Manufactory Co., Ltd., Xiamen, China) was adopted to determine the LPS endotoxin in the rumen fluid, as introduced in former investigations [33, 34].

The AI-2 signal was quantified on a 1260 high performance liquid chromatography equipped with a fluorescence detector (HPLC-FD) system (Agilent Technologies, Santa Clara, USA) using a ZORBAX Eclipse XDB-C18 column (250 mm × 4.6 mm, 5 μm; Agilent, Santa Clara, USA), in accordance with the protocols depicted in earlier reports [14, 35]. For the detection of AHLs including C4-homoserine lactone (HSL), C6-HSL, C8-HSL, C10-HSL, 3-oxo-C6-HSL, 3-oxo-C8-HSL, 3-oxo-C10-HSL, and 3-oxo-C12-HSL, the ExionLC 30A-QTRAP 5500 ultra-high performance liquid chromatographymass spectrometry (UHPLC-MS) system (AB SCIEX, Framingham, USA) with an ACQUITY BEH C18 column (2.1 mm × 100 mm) (Waters Corporation, Milford, USA) was employed using the procedures developed by Doberva et al. [36] and Wang et al. [14].

### Full-length 16S rRNA gene sequencing analysis

Firstly, the genomic DNA was isolated from the rumen liquid samples through previously described approaches [14, 37]. By adopting the barcoded universal primers 27 F (50-AGRGTTTGATYNTGGCTCAG-30) and 1492R (50-TASGGHTACCTTGTTASGACTT-30), the full-length 16S rRNA genes were then amplified, followed by the amplicons quantitation and combination [37]. The amplicon sequencing library was constructed using the SMRTbell prep kit 3.0 (Pacific Biosciences, MenloPark, USA) as per the manufacturer’s manual, and then sequenced on the PacBio Sequel IIe platform (Pacific Biosciences, MenloPark, USA) with single-end reads generated. Subsequently, the circular consensus sequencing (CCS) reads recognition, CCS reads quality filtering, and chimera sequence removal were accomplished. The SILVA database (release 138) [38] was adopted to fulfill the operational taxonomic unit (OTU) taxonomic annotation with a confidence threshold at 97%, and OTU abundance normalization was performed using a standard of sequence number corresponding to the sample with the least sequences. Subsequently, the Alpha diversity and Beta diversity were analyzed via the QIIME (version 1.9.1) and R software (version 3.5.0) [14, 31, 37]. All the raw sequences generated in this experiment were deposited to the sequence read archive (SRA) of the NCBI database under the accession number PRJNA1250112.

### Metabolomics analysis

As described in detail in prior studies [14, 39], the metabolomic analysis targeting the rumen fluid was carried out, consisting of the metabolites extraction, UHPLC-MS analysis, raw data treatment, metabolites annotation, and metabolomics data analysis. Those metabolites respectively identified under the positive and negative polarity mode were mixed, and then the principal components analysis (PCA) and partial least squares discriminant analysis (PLS-DA) were carried out ahead of the internal standard normalization and data logarithmic transformation. The metabolic pathway classification and enrichment analysis were completed via the MetaboAnalyst (version 5.0) [40] based on the KyotoEncyclopedia of Genes and Genomes (KEGG) database (release 110.1).

### Statistical analysis

The GLM procedure of SAS (V9.4, SAS Institute Inc., Cary, USA) was employed to assess the influences of ZEN on the fermentation characteristics, nutrient disappearance rates, LPS concentration, QS signals, and Alpha diversity indexes, through the following statistical model:

$$Y_{ijkl}=\mathrm\mu+P_i+S_{\mathrm l}+V_{j(l)}+T_k+S_{Tlk}+{\mathrm\varepsilon}_{ijkl}$$

where *Y*_*ijkl*_ is the dependent variable, *μ* is the overall mean, *P*_*i*_ is the fixed effect of period *i*, *S*_*l*_ is the fixed effect of square *l* *(l* = 1, 2 or 3), V_*j*(*l*)_ is the fixed effect of vessel *j* within square *l*, *T*_*k*_ is the fixed effect of ZEN supplementation treatment *k*, *S*_*Tlk*_ is the fixed interaction effect between square *l* and treatment *k*, and ε_*ijkl*_ is the random residual error. The observed means were compared through the Tuckey’s HSD test, and the linear and quadratic effects of the ZEN dosage was checked using the orthogonal polynomial contrast with the coefficients generated by the IML procedure. Statistical difference was respectively declared as significant, highly or extremely significant with *P* < 0.05, < 0.01 or < 0.001, and trend was considered at 0.05 < *P* ≤ 0.10. The significant differences in the relative abundances of bacterial taxa were examined and visualized through the linear discriminant analysis effect size (LEfSe) analysis with a linear discriminant analysis (LDA) score > 2.0 and *P* < 0.05. Significantly differential potential functions of the bacterial microbiota were predicted via the Kruskal–Wallis test at *P* < 0.05, based on the phylogenetic investigation of communities by reconstruction of unobserved states 2 (PICRUSt2) analysis (release v2.6.2) [41]. The significantly different metabolites between or across treatments were respectively classified by the unpaired *t*-test or the Kruskal–Wallis test, with the variable importance in projection (VIP) value > 1 and *P* < 0.05. The model-based integration of metabolite observations and species abundances 2 (MIMOSA2, version 2.1.0) analysis [42] was employed to inquire into the potential interconnections between bacterial species and the differential metabolites, by using the bacterial species abundance data and comparing the community-level metabolic potential scores with the metabolite measurements in a linear regression model via the R software (version 3.5.0). The Spearman correlation analysis was conducted to explore the interrelationships between the QS signals, differential fermentation parameters, differential bacterial taxa, and differential metabolites.

## Results

### QS signals, fermentation characteristics, and nutrient disappearances

The C4-HSL and 3-oxo-C8-HSL molecules were the only two AHLs detected in the present experiment. The ZEN exposure significantly (*P* < 0.05) reduced the level of AI-2, and tended to lower the C4-HSL (*P* = 0.051) in the rumen liquid (Table 2). Quadratic declines of the total volatile fatty acid (TVFA; *P* < 0.001), acetate (*P* < 0.001), isobutyrate (*P* < 0.05), and isovalerate (*P* < 0.001) under the ZEN challenge were respectively observed, while the propionate linearly (*P* < 0.05) decreased in response to ZEN inclusion. It was noteworthy that the LPS concentration in the ZENH was significantly (*P* < 0.05) reduced when compared with the CON. Besides, the organic matter disappearance (OMD), neutral detergent fiber disappearance (NDFD), and acid detergent fiber (ADFD) were qudratically (*P* < 0.001) depressed by ZEN.

**Table 2:** Responses of quorum sensing signal molecules, RUSITEC fermentation characteristics, and disappearance rates of nutrients to the ZEN challenge

Item^1^	Treatment^2^				SEM^3^	*P*-value^4^		
	CON	ZENL	ZENM	ZENH		Dose	L	Q
Quorum sensing signal molecules								
AI-2, ng/mL	5.53^a^	4.63^b^	4.20^bc^	4.05^c^	0.090	< 0.001	0.009	0.033
C4-HSL, pg/mL	100.70	75.80	57.88	72.28	8.185	0.051	0.021	0.031
3-oxo-C8-HSL, pg/mL	13.75	11.65	11.98	31.75	9.673	0.453	0.751	0.667
Fermentation parameters								
pH	6.70	6.71	6.69	6.70	0.024	0.874	0.497	6.70
NH_3_-N, mmol/L	0.20	0.21	0.21	0.21	0.002	0.209	0.618	0.353
TVFA, mmol/L	120.0^a^	124.1^a^	115.9^a^	98.6^b^	3.13	< 0.001	< 0.001	< 0.001
Acetate, mmol/L	87.4^ab^	89.9^a^	84.2^b^	71.8^c^	2.01	< 0.001	< 0.001	< 0.001
Propionate, mmol/L	21.2^a^	21.9^a^	20.1^ab^	18.5^b^	0.94	0.013	0.020	0.071
Butyrate, mmol/L	8.11	8.54	7.83	6.82	0.576	0.058	0.029	0.087
Isobutyrate, mmol/L	1.05^ab^	1.37^a^	1.06^ab^	0.83^b^	0.184	0.026	0.004	0.019
Valerate, mmol/L	1.35	1.54	1.53	1.18	0.088	0.269	0.470	0.535
Isovalerate, mmol/L	0.74^a^	0.81^a^	0.72^a^	0.58^b^	0.035	0.015	0.001	0.005
A:P	4.16	4.10	4.13	3.98	0.162	0.793	0.378	0.646
MCP, mg/mL	0.56	0.60	0.60	0.60	0.019	0.313	0.865	0.405
LPS, EU/mL	48,011^a^	44,290^ab^	53,910^a^	28,851^b^	6,141.6	0.013	0.416	0.575
Nutrients disappearances, %								
DMD	74.06	72.15	68.57	68.26	2.437	0.269	0.007	0.021
OMD	72.28^a^	71.57^b^	70.82^c^	68.01^d^	0.086	< 0.001	< 0.001	< 0.001
CPD	83.78	83.92	84.57	83.04	0.491	0.154	0.173	0.143
NDFD	66.35^a^	64.35^b^	63.00^b^	60.26^c^	0.381	< 0.001	< 0.001	< 0.001
ADFD	64.18^a^	61.19^b^	59.49^b^	57.20^c^	0.673	< 0.001	< 0.001	< 0.001

^a–c^Means within a row for treatments that do not have a common superscript differ at *P* < 0.05

^1^*AI-2 *Autoinducer-2, *TVFA *Total volatile fatty acid, *A:P *The ratio of acetate to propionate, *MCP *Microbial protein, *LPS *Lipopolysaccharide, *EU *Endotoxin unit, *DMD *Dry matter disappearance, *OMD *Organic matter disappearance, *CPD *Crude protein disappearance, *NDFD *Neutral detergent fiber disappearance, *ADFD *Acid detergent fiber disappearance, *ZEND *ZEN disappearance

^2^*CON *Control, *ZENL *Low-dose ZEN treatment, *ZENM *Mid-dose ZEN treatment, *ZENH *High-dose ZEN treatment

^3^*SEM* Standard error of means for treatments

^4^*L *Linear effect of the ZEN dose, *Q *Quadratic effect of the ZEN dose

### Ruminal bacterial microbiome

**Fig. 1:**
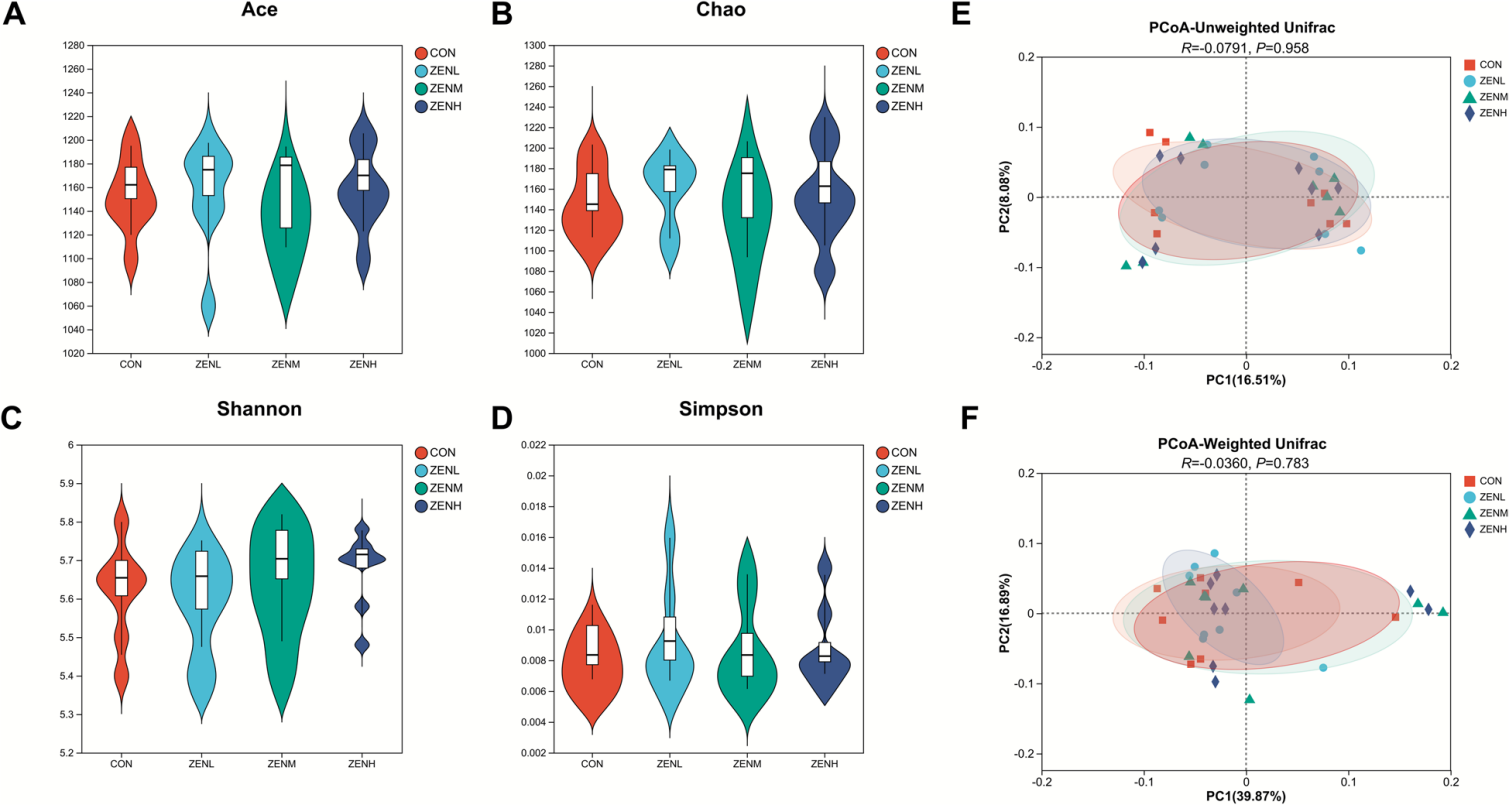
Effects of ZEN at different doses on the Alpha diversity indices and Beta diversity. **A** Ace index. **B** Chao index. **C** Shannon index. **D** Simpson index. **E** Principal coordinate analysis (PCoA) profile based on the unweighted Unifrac matrix. **F** PCoA profile based on the weighted Unifrac matrix

**Fig. 2:**
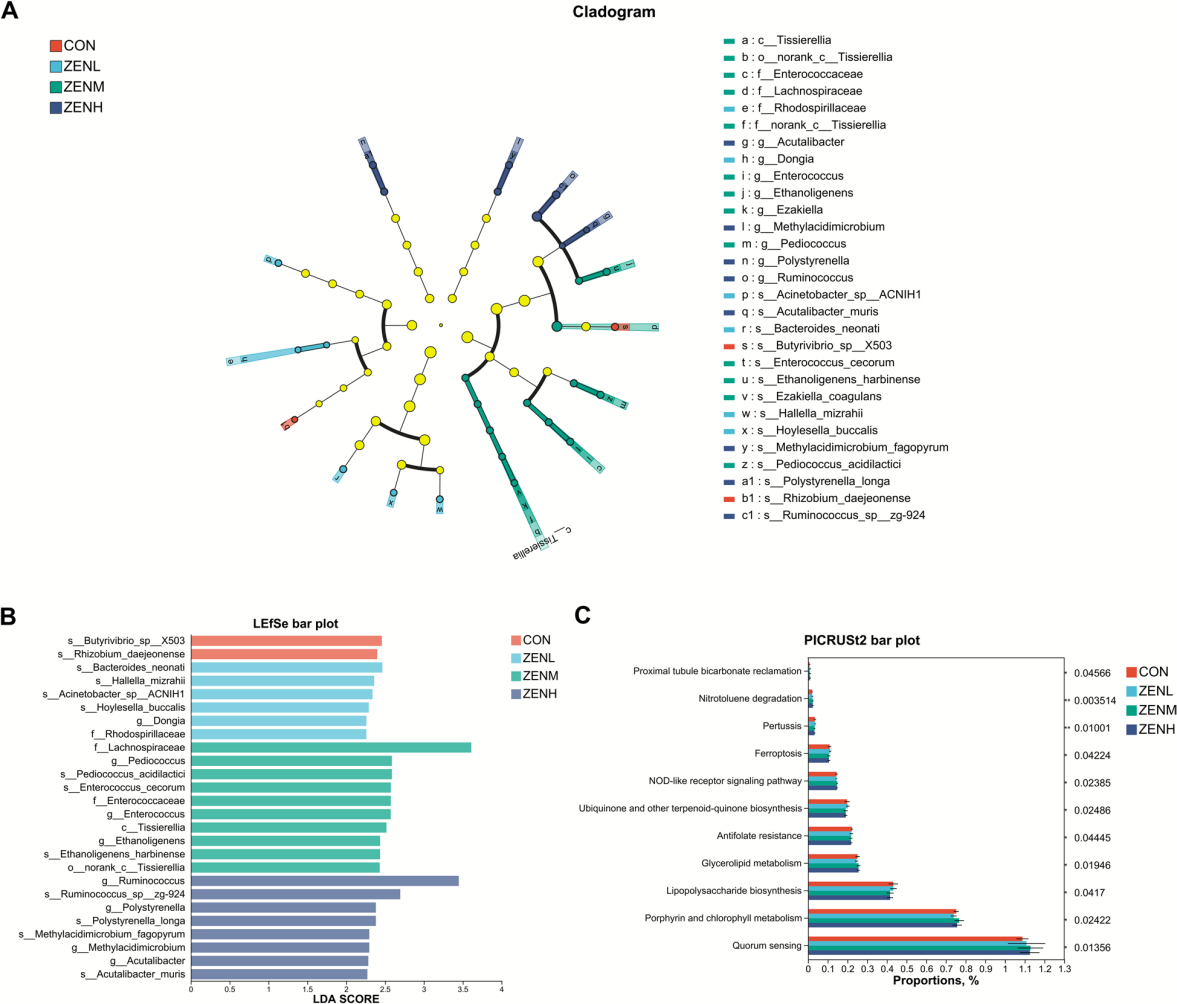
Effects of ZEN at different doses on the bacterial taxa relative abundances, and the predicted functions of the ruminal bacterial microbiota. **A** Cladogram displaying significantly enriched bacterial taxa (LDA score > 2.0 and *P* < 0.05) from the phylum to the species level through the LEfSe analysis. **B** LEfSe bar chat displaying the significantly differential taxa across different treatments. The LDA scores represented the difference in relative abundance with exponent fold change of 10 across treatments. **C** Kruskal–Wallis test for the predicted potential functions for the ruminal bacterial microflora across treatments based on the PICRUSt2 analysis

In the present study, no significant (*P* > 0.05) differences in the Alpha diversity indices across treatments was shown (Fig. 1A–D, Table S1). As was illustrated in Fig. 1E and F, neither unweighted nor weighted Unifrac-based principal coordinate analysis (PCoA) showed treatment-dependent clustering of the bacterial populations. The LEfSe charts (Fig. 2 A and B, Tables S2 and S3) suggested that the species of *Butyrivibrio_sp_X503* and *Rhizobium daejeonense* were significantly (*P* < 0.05) abundant in the CON, whilst the family Rhodospirillaceae and its affiliated genus *Dongia*, along with the species *Bacteroides neonati*, *Hallella mizrahii*, *Acinetobacter_sp_ACNIH1*, and *Hoylesella buccalis* were annotated with significantly (*P* < 0.05) higher abundances in the ZENL than the CON. The relative abundances of the bacterial class Tissierellia and its lower order norank*_*c*_*Tissierellia and family norank_c_Tissierellia, as well as the further subordinate *Ezakiella* and *Ezakiella coagulans* were sequentially significantly (*P* < 0.05) raised in the ZENM group. In addition, the significant (*P* < 0.05) enrichments of Enterococcaceae, *Enterococcus*, and *Enterococcus cecorum* in ZENM were marked. Further, *Pediococcus* and *Pediococcus acidlactici*, *Ethanoligenens* and *Ethanoligenens harbinense*, and the family Lachnospiraceae were identified as significantly (*P* < 0.05) enriched taxa in ZENM. As for the ZENH treatment, from the genus level to the species level, the *Ruminococcus* and *Ruminococcus_sp_zg-924*, *Polystyrenella* and *Polystyrenella longa*, *Methylacidimicrobium* and *Methylacidimicrobium fagopyrum*, *Acutalibacter* and *Acutalibacter muris* were classified with significantly (*P* < 0.05) more abundances. According to the PICRUSt2 analysis (Fig. 2 C), QS, porphyrin and chlorophyll metabolism, LPS biosynthesis, glycerolipid metabolism, and other 7 potential functions of the bacterial community were predicted to be significantly (*P* < 0.05) affected by the ZEN contamination.

### Rumen metabolome

**Fig. 3:**
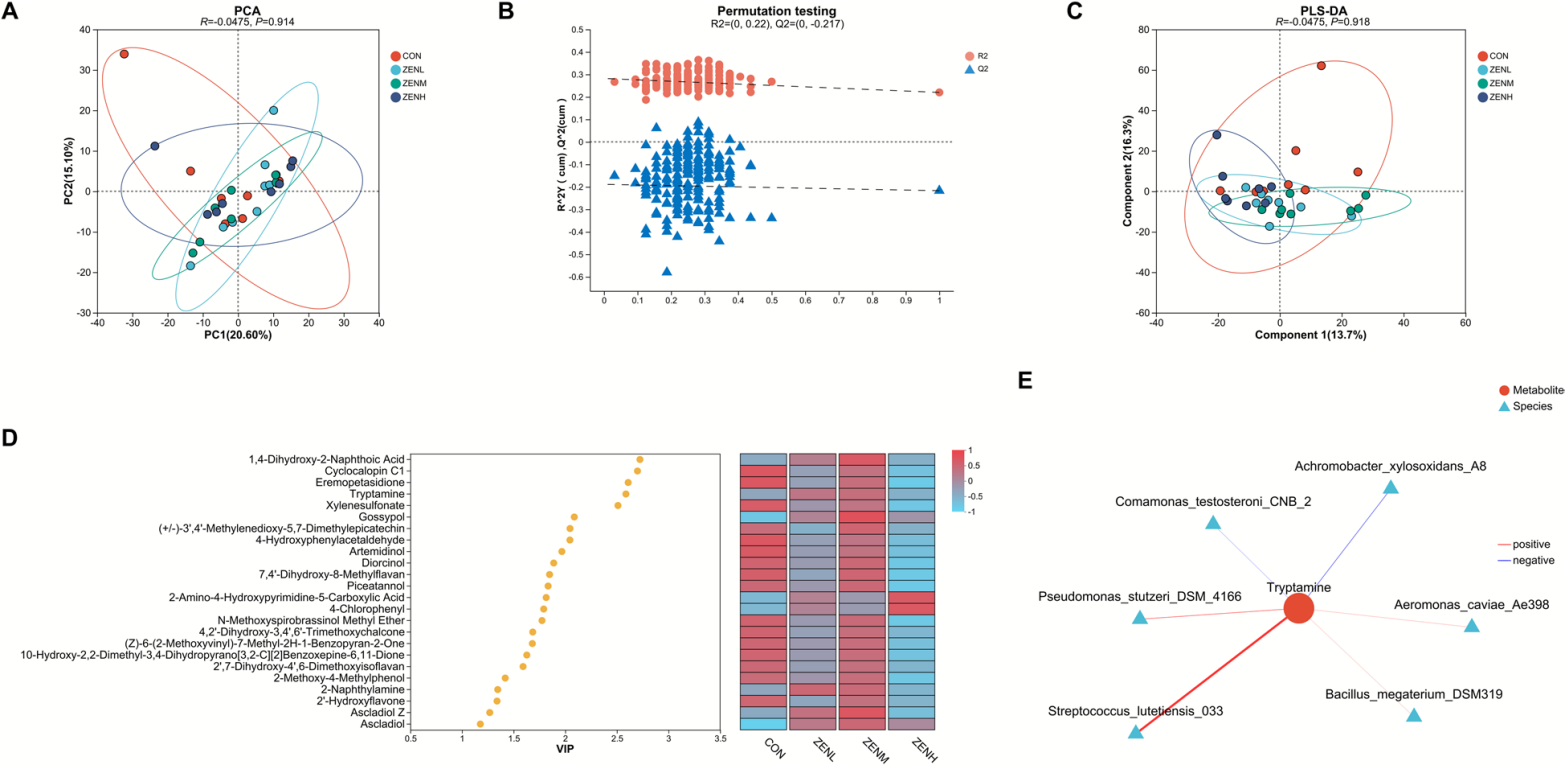
Effects of ZEN at different doses on the ruminal metabolomic profiles through comprehensive analysis. **A** Principal component analysis (PCA) score scatter plot. **B** Permutation testing chart for the principal coordinate analysis (PLS-DA). **C** PLS-DA score scatter plot. **D** Variable importance in projection (VIP) bubble plot for the commonly significantly (*P* < 0.05) differential metabolites across all the treatments. **E** Model-based integration of metabolite observations and species abundances 2 (MIMOSA2) analysis depicting the potential correlations between the significantly (*P* < 0.05) differential metabolites and bacterial species

**Fig. 4:**
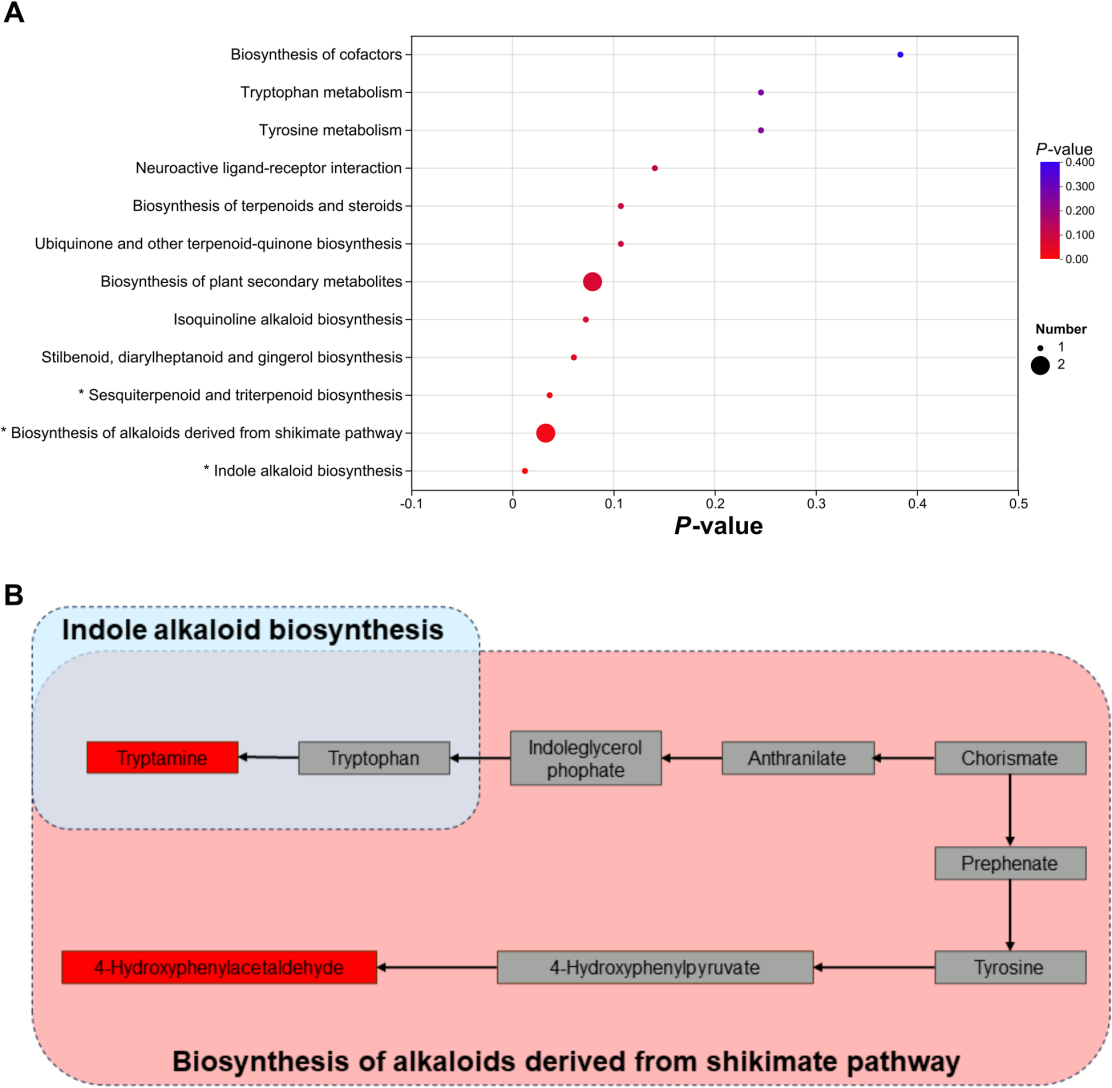
Effects of ZEN at different doses on the ruminal metabolic pathways through comprehensive analysis. **A** Bubble plot for the KEGG enrichment analysis of the commonly differential metabolic pathways across treatments. The size of the bubble represents the number of the enriched differential metabolites for each pathway. The asterisk indicates that the corresponding metabolic pathway is detected as significantly (*P* < 0.05) differential across treatments. **B** Integrated significantly (*P* < 0.05) differential metabolic pathways between treatments. The metabolites in the red boxes are identified as significantly (*P* < 0.05) differential

The PCA plot (Fig. 3A) displayed no clear distinction for the metabolic patterns across treatments, neither did the PLS-DA diagram (Fig. 3C) which was validated through the permutation test (Fig. 3B). However, evident separations in the metabolomic profiles between treatments were portrayed by the PLS-DA charts based on pairwise comparisons (Fig. S1), and the significantly (*P* < 0.05) differentially expressed metabolites between treatments were listed in Table S4 and Fig. S2. A total of 24 metabolites, such as 1,4-dihydroxy-2-naphthoic acid, cyclocalopin C1, eremopetasidione, and tryptamine, were detected as commonly significantly (*P* < 0.05) different metabolites across all the treatments (Fig. 3D, Table S5). It was revealed by the MIMOSA2 analysis (Fig. 3E) that *Streptococcus lutetiensis_033*, *Pseudomonas stutzeri_DSM_4166*, *Bacillus megaterium_DSM319*, and *Aeromonas caviae_Ae398* were positively correlated with the tryptamine, while the negative interconnections of this differential metabolite with *Achromobacter xylosoxidans_A8* and *Comamonas testosteroni_CNB_2* were also exhibited. As was demonstrated by the KEGG enrichment based on the commonly differential metabolite identification, 12 metabolic pathways were annotated as differential across treatments, amongst which the indole alkaloid biosynthesis, biosynthesis of alkaloids derived from shikimate pathway, and sesquiterpenoid and triterpenoid biosynthesis significantly (*P* < 0.05) differed across treatments (Fig. 4A, Table S6). The indole alkaloid biosynthesis and biosynthesis of alkaloids derived from shikimate pathway were further integrated with reference to the KEGG maps, with the relevant differing metabolites being highlighted in Fig. 4B. The significantly different metabolic pathways and corresponding differential abundance score plots through pairwise contrasts were presented in Fig. S2 and Table S7.

### Correlation analysis

**Fig. 5:**
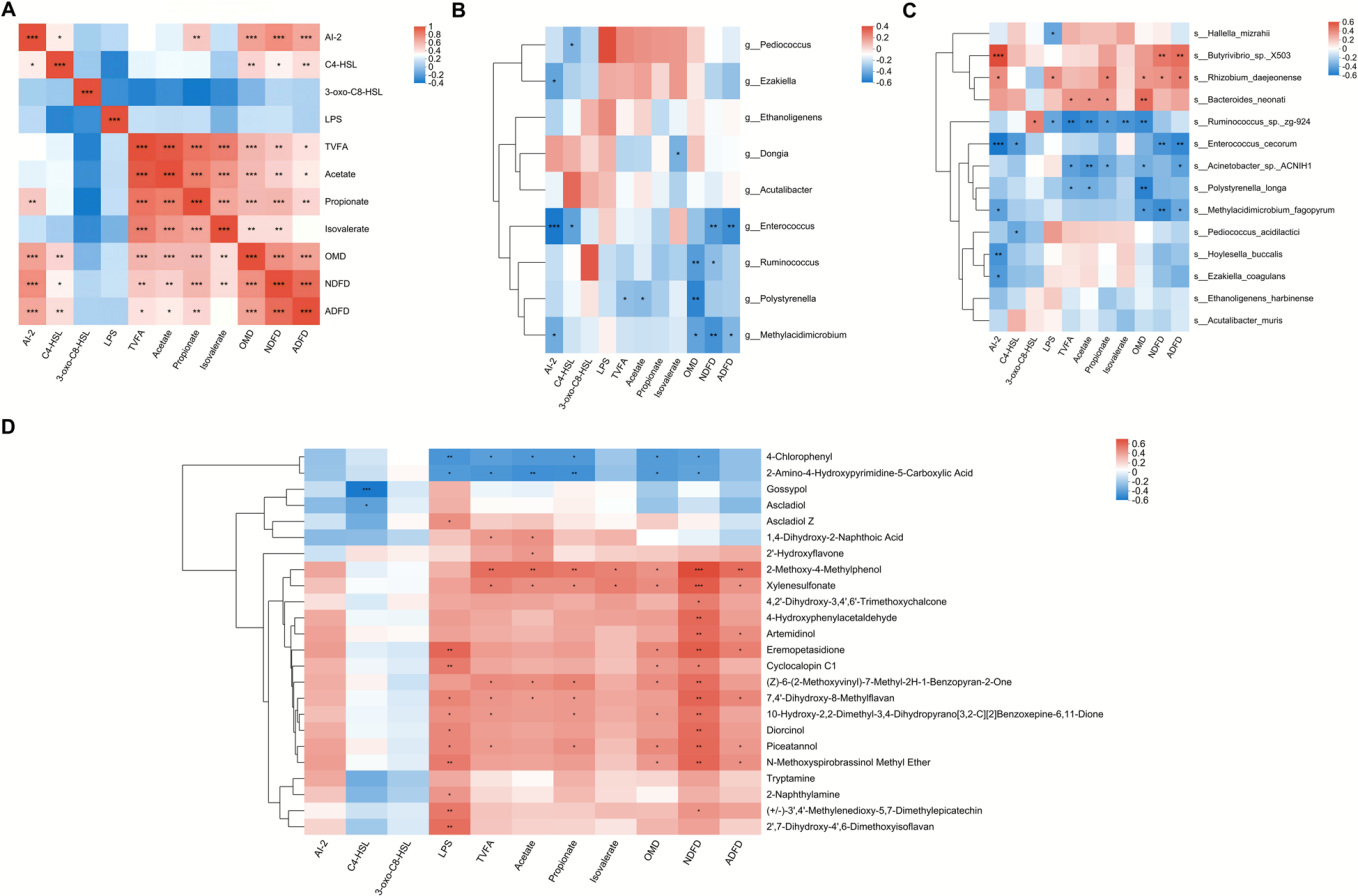
Correlations between significantly (differential QS molecules and rumen fermentation traits, and correlations of significantly differential QS molecules and rumen fermentation traits with significantly differential bacterial genera, bacterial species, and ruminal metabolites demonstrated through Spearman analysis. **A** Correlations between significantly (*P* < 0.05) differential QS molecules and rumen fermentation traits. **B** Correlations of significantly (*P* < 0.05) differential QS molecules and rumen fermentation traits with significantly (*P* < 0.05) differential bacterial genera. **C** Correlations of significantly (*P* < 0.05) differential QS molecules and rumen fermentation traits with significantly (*P* < 0.05) differential bacterial species. **D** Correlations of significantly (*P* < 0.05) differential QS molecules and rumen fermentation traits with significantly (*P* < 0.05) differential ruminal metabolites

It was depicted by the Spearman analysis that the AI-2 signal significantly positively correlated with the propionate (*P* < 0.01), OMD (*P* < 0.001), NDFD (*P* < 0.001), and ADFD (*P* < 0.001), and the highly significantly (*P* < 0.01) positive connections of C4-HSL with OMD and NDFD were witnessed (Fig. 5A). Besides, positive associations amongst TVFA, acetate, propionate, isovalerate, OMD, NDFD, and ADFD to varying degrees were also demonstrated. For the differential genera, it was found that, at different levels, the QS signals of AI-2 and C4-HSL negatively (*P* < 0.05) interacted with *Pediococcus*, *Ezakiella*, *Enterococcus*, or *Methylacidimicrobium* (Fig. 5B). Negative (*P* < 0.05) correlations of TVFA, acetate, isovalerate, OMD, NDFD, and ADFD with *Dongia*, *Enterococcus*, *Ruminococcus*, *Polystyrenella*, or *Methylacidimicrobium* were also displayed to different extents. In terms of the differing species, *Butyrivibrio_sp_X503* (*P* < 0.001) and *Rhizobium daejeonense* (*P* < 0.01) were positively connected with AI-2, while the negative interrelationships of *Enterococcus cecorum* (*P* < 0.001), *Methylacidimicrobium fagopyrum* (*P* < 0.05), *Hoylesella buccalis* (*P* < 0.01), and *Ezakiella coagulans* (*P* < 0.05) with AI-2 were showed simultaneously (Fig. 5C). Further, *Enterococcus cecorum* and *Pediococcus acidilactici* both significantly (*P* < 0.05) negatively interacted with C4-HSL, whilst *Ruminococcus_sp_zg-924* was positively related with 3-oxo-C8-HSL (*P* < 0.05). *Rhizobium daejeonense* was found to positively (*P* < 0.05) contribute to the LPS, but *Hallella mizrahii* and *Ruminococcus_sp_zg-924* both negatively (*P* < 0.05) correlated to LPS. Moreover, *Butyrivibrio_sp_X503*, *Rhizobium daejeonense*, and *Bacteroide neonati* were basically positively correlated with TVFA, acetate, propionate, OMD, NDFD, or ADFD to varying extents. By contrast, the general negative interplays of *Ruminococcus_sp_zg-924*, *Enterococcus cecorum*, *Acinetobacter_sp_ACNIH1*, *Polystyrenella longa*, and *Methylacidimicrobium fagopyrum* with the abovementioned parameters to different degrees were manifested.

**Fig. 6:**
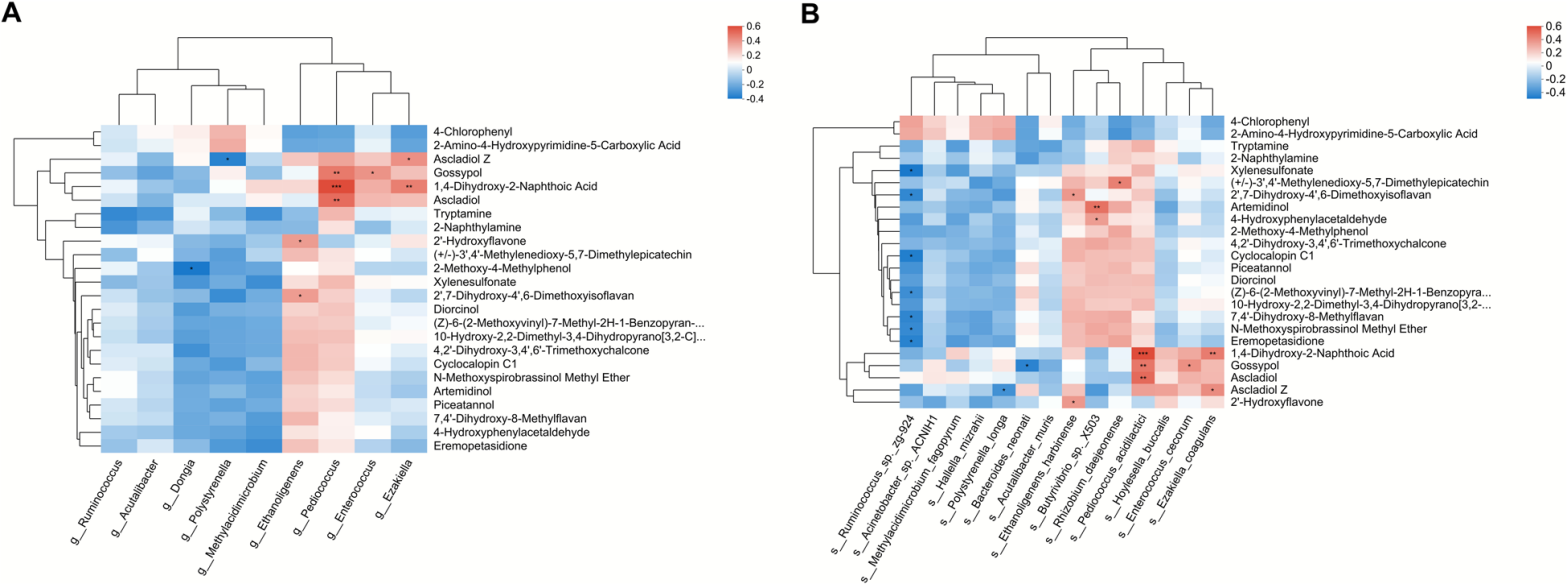
Correlations of significantly differential ruminal metabolites with significantly differential bacterial genera and bacterial species demonstrated through Spearman analysis. **A** Correlations between significantly (*P* < 0.05) differential bacterial genera and significantly (*P* < 0.05) differential ruminal metabolites. **B** Correlations between significantly (*P* < 0.05) differential bacterial species and significantly (*P* < 0.05) differential ruminal metabolites

In regard of the commonly differential metabolites, the gossypol (*P* < 0.001) and ascladiol (*P* < 0.05) were found to negatively correlate with the C4-HSL signal (Fig. 5D). The negative interactions of 4-chlorophenl (*P* < 0.01) and 2-amino-4-hydroxypyrimidine-5-carboxylic acid (*P* < 0.05) with LPS, and the positive correlations of ascladiol Z (*P* < 0.05), eremopetasidione (*P* < 0.01), cyclocalopin C1 (*P* < 0.01), and other 8 differing metabolites with LPS were concurrently shown. In brief, 4-chlorophenl and 2-amino-4-hydroxypyrimidine-5-carboxylic acid negatively correlated with the majority of the differing fermentation traits and nutrient disappearance rates, while a series of distinctive metabolites such as the 2-methoxy-4-methylhenol and xylenesulfonate were found to be positively connected to those forementioned differing parameters at different levels. As to the differential bacterial genera and metabolites, the significantly (*P* < 0.05) negative interplays between *Dongia* and 2-methoxy-4-methylphenol, as well as *Polystyrenella* and ascladiol Z were noted (Fig. 6A). Additionally, *Ethanoligenens*, *Pediococcus*, *Enterococcus*, and *Ezaliella* were discovered to positively relate to several differing metabolites to varying degrees. At the species level, *Ruminococcus_sp_zg-924*, *Polystyrenella longa*, and *Bacteroide neonati* were significantly (*P* < 0.05) negatively connected to some of the different metabolites, despite the various degrees of positive (*P* < 0.05) associations of *Ethanoligenens harbinense*, *Butyrivibrio_sp_X503*, *Rhizobium daejeonense*, *Pediococcus acidlactici*, *Enterococcus cecorum*, and *Ezakiella coagulans* with a few distinctive metabolites (Fig. 6B).

## Discussion

In the current study, the negative impacts of ZEN supplementation on the QS signals of AI-2 and C4-HSL, along with the prediction that QS would be affected by ZEN through PICRUSt2 analysis, supported the hypothesis that QS might be involved in the responses of ruminal microbes to ZEN exposure. Hartinger et al. [4] reported the declines of both ruminal pH and total short-chain fatty acids in cows subjected to 5 mg of ZEN per day. In comparison, despite the unaffectedness of pH and DMD observed in this experiment, ZEN contamination reduced the ruminal densities of TVFA, acetate, propionate, isobutyrate, isovalerate, and lowered OMD, NDFD, and ADFD in different manners, indicating that ZEN might impair rumen fermentation mainly through suppressing the fiber digestion. These findings together confirmed that even though ZEN can be decomposed by the rumen bacteria to a certain extent [43, 44], it would still exert detrimental influences on the ruminal bacterial fermentation. Further, the positive correlations of AI-2 and C4-HSL with propionate, OMD, NDFD, or ADFD depicted through the Spearman analysis of this trial were in line with the viewpoint that QS could play a nonnegligible role in modulating the feed degradation and rumen fermentation patterns [7, 16], implying that both the AI-2-mediated and AHLs-based QS might participate in the impairments of rumen fermentation dynamics by ZEN. Moreover, the abating effect of ZEN on the ruminal LPS was demonstrated via both the LPS determination and PICRUSt2 prediction, indicating that ZEN can interfere with the LPS production by ruminal bacteria. It could be speculated that, based on the evidences about the regulatory activities of QS in the LPS generation of typical Gram-native bacteria [45–47], the LPS decrement could be related to the synchronously declining trends of AI-2 and C4-HSL by ZEN. However, further investigations are warranted to testify this assumption.

The potential involvements of QS during the bacterial variations by ZEN exposure in this experiment were further disclosed through 16S rRNA gene sequencing analysis combined with the Spearman analysis. As a priorly acknowledged constituent of the commensal microflora inhabiting gastrointestines, *Enterococcus cecorum* has emerged as an antimicrobial-resistant pathogen for the global poultry industry [48, 49]. Therefore, the enrichment of *Enterococcus cecorum* in the rumen liquid of ZENM group might stem from its antimicrobial-resistance and potentially induce health risks for the host ruminants. Further, the negative interconnections of the *Enterococcus* and *Enterococcus cecorum* abundances with AI-2 and C4-HSL were discovered in this trial. In contrast, it had been separately reported that the proliferation of another pathogenic member of the genus *Enterococcus*, i.e., *Enterococcus faecailis*, was unaltered by either *luxS* gene depletion [50] or AHLs exposure [51]. This discrepancy between these two species requires further research to be deciphered.

The respective enrichments of *Ezakiella coagulans*, *Hoylesella buccalis*, and *Methylacidimicrobium fagopyrum* under the ZEN challenge, as well as their negative interrelations with the AI-2 signal were witnessed concurrently in this study. *Ezakiella coagulans* is an obligate anaerobe previously discovered in human vagina [52] and stallion semen [53], while *Hoylesella buccalis* is a prevalent colonizer in human vagina and oral cavity that used to be referred as *Prevotella buccalis* [54, 55], yet little is known about their exact roles in the ruminal bacterial consortia and QS systems at present. Considering the methanotrophic property of *Methylacidimicrobium fagopyrum* [56], it could be inferred that the increment of this taxon in ZENH would attenuate the methane yield during rumen fermentation, which necessitates further experiments to be checked. As a lactic acid bacterium, *Pediococcus acidilactici* has been identified with the existence of *luxS* gene in its core genome [57]. In comparison, this trial revealed that *Pediococcus acidilactici* was negatively related to the C4-HSL molecule, implying the complicacy of the QS systems and the necessity of deeper explorations.

Since *Butyrivibrio_sp_X503* has been characterized as a ruminal lignocellulolytic species with acetate being its primary fermentation product [58], thence its abundance decline in the ZEN-supplemented groups could serve as a partial explanation for the synchronous decreases in TVFA, acetate, NDFD, and ADFD, which was also consistent with its positive correlations with NDFD and ADFD marked in this trial, as well as the above-introduced assumption that ZEN might interrupt rumen fermentation mainly through repressing fiber degradation. Besides, the positive relation between *Butyrivibrio_sp_X503* and AI-2 in this study was also in line with the high abundances of the *LuxS*-relevant AI-2 QS genes presented by *Butyrivibrio* spp. in prior report [5]. The ubiquitousness of AHLs-dependent QS system in *Rhizobium* spp. has been recorded formerly [59, 60], while this study noted the positive interconnections of the Gram-negative species *Rhizobium daejeonense* [61] with AI-2, LPS, propionate, OMD, NDFD, and AFDF, as well as the abundance reduction of this taxon by ZEN exposure. These results collectively not only indicated that the abatement of *Rhizobium daejeonense* might to some extent contribute to the decreases in OMD, NDFD, and AFDF, but also conveyed the potential significance of *Rhizobium daejeonense* for the AI-2-mediated QS, LPS production, and ruminal microbial fermentation in response to ZEN contamination. However, this supposition requires deeper studies to be examined.

Won et al. [5] noted that *Ruminococcus* spp. seized highly abundant AI-2-related QS genes within the rumen microbiome, whilst positive relation between *Ruminococcus_sp_zg-924* and 3-oxo-C8-HSL was found in this trial. Additionally, the negative interrelations of *Ruminococcus_sp_zg-924* with some VFAs seemed to be contradictory to an earlier research that discovered the positive correlations of *Ruminococcus* with acetate, butyrate, and TVFA [62]. These distinctions provided novel finding on the engagement of *Ruminococcus* spp. in AHLs-mediated QS, and warranted further investigations to elucidate the effects of interactions between *Ruminococcus* and QS on ruminal microbial fermentation under ZEN challenge. Moreover, the enrichment of *Acinetobacter_sp_ACNIH1* and *Polystyrenella longa* by ZEN suggested these two taxa might adapt to the ZEN exposure, and their negative correlations with the fermentation traits implied that their abundance rises could contribute to the general suppression of rumen fermentation by ZEN contamination.

In the present study, plenty of differential metabolites were annotated through the pairwise contrasts as well as the overall analysis across all the treatments, which offered abundant repository to the searching for efficient biomarkers signifying the ZEN toxicity on ruminal microbial fermentation. According to the Spearman analysis, it was further demonstrated that the differing metabolite gossypol negatively correlated with the C4-HSL signal. Since Noumi et al. [63] also noticed the anti-QS effect of gossypol as a major constituent of the *Thymus musilii* Velen. metanolic extract, it might thereby be inferred that gossypol could be exploited as a quorum quenching (QQ) agent for the AHLs-dependent QS. Moreover, the positive interconnections of both *Pediococcus acidilactici* and *Enterococcus cecorum* with gossypol were in agreement with the above-discussed correlations of these two species with C4-HSL, further indicating the potential roles of these species in interrupting AHLs-mediated QS. Noticeably, a QQ bacterium named HEMM-1 belonging to the *Enterococcus* spp. was found to hydrolyze AHLs with the aid of its lactonase activity [64]. Therefore, in combination with the evidences concerning the significance of lactonases in ZEN degradation via hydroxylating the lactone bond of ZEN [65, 66], it could be assumed that *Enterococcus cecorum* and other bacteria that might possess lactonase property, could thence thrive under the ZEN challenge, with the AHLs-based QS being disrupted simultaneously [67]. This speculation was supported by the abovementioned enrichment of *Enterococcus cecorum* in the ZEN-added treatments, but it requires deeper experiments to be validated. In addition, the negative bond between the differential metabolite ascladiol and C4-HSL, as well as the respectively negative connections of *Pediococcus acidilactici* and *Ezakiella coagulans* with ascladiol or ascladiol Z (stereoisomer of ascladiol) were observed in this study. The ascladiol has been commonly identified as a main biodegradation product of another mycotoxin patulin [68], but no information about the relation between this chemical and the ZEN metabolism is accessible currently.

In accordance to the KEGG enrichment analysis, the indole alkaloid biosynthesis, biosynthesis of alkaloids derived from shikimate pathway, and sesquiterpenoid and triterpenoid biosynthesis, that are all relevant to QS systems, were annotated as significantly differential metabolic pathways across all the treatments. By searching in 2,809 metagenome-assembled genomes from the rumen microbiota, Liu et al. [10] reported that 103 ruminal bacterial species used indoles as interkingdom and interspecies signaling molecules, amongst which 79 species exhibited the *tnaA* gene encoding tryptophanase that catalyzes the synthesis of indoles. More specifically, indoles had been found to suppress the AHLs-based QS communication of a number of representative bacteria [69, 70], which could help to associate the differentiation of indole alkaloid biosynthesis with the tendentious decrement in AHLs of this trial. Further, based on the MIMOSA2 analysis, it could be inferred that those bacterial species differently related to the differing metabolite tryptamine might be involved in the QS changes by ZEN, as tryptamine acts as the fundamental chemical and biosynthetic link between simple molecule indole and complex indole alkaloids [71].

As a metabolic pathway bound up with the indole alkaloid biosynthesis, the biosynthesis of alkaloids derived from shikimate pathway could be inferred to be correlated to the QS variation by the ZEN contamination in this experiment as well. Besides, Kang et al. [72] found that the QS system within the bacterial communities of chicken manure could manipulate the metabolisms of carbohydrates and amino acids through affecting the shikimate pathway, while the upregulation of shikimate pathway by the C14-HSL signal in a typical pathogen *Trichosporon asahii* was marked by Lu et al. [73]. For the sesquiterpenoid and triterpenoid biosynthesis, its role in the QS changes induced by ZEN in the present investigation was also supported by prior studies. For instance, as a common sesquiterpene, farnesol is generated by the opportunistic fungal pathogen *Candida albicans* as a QS molecule to regulate the infection dynamics [74]. In addition, many members of the triterpenoids have been characterized with antimicrobial effects and QS-inhibitory activities [75].

**Fig. 7:**
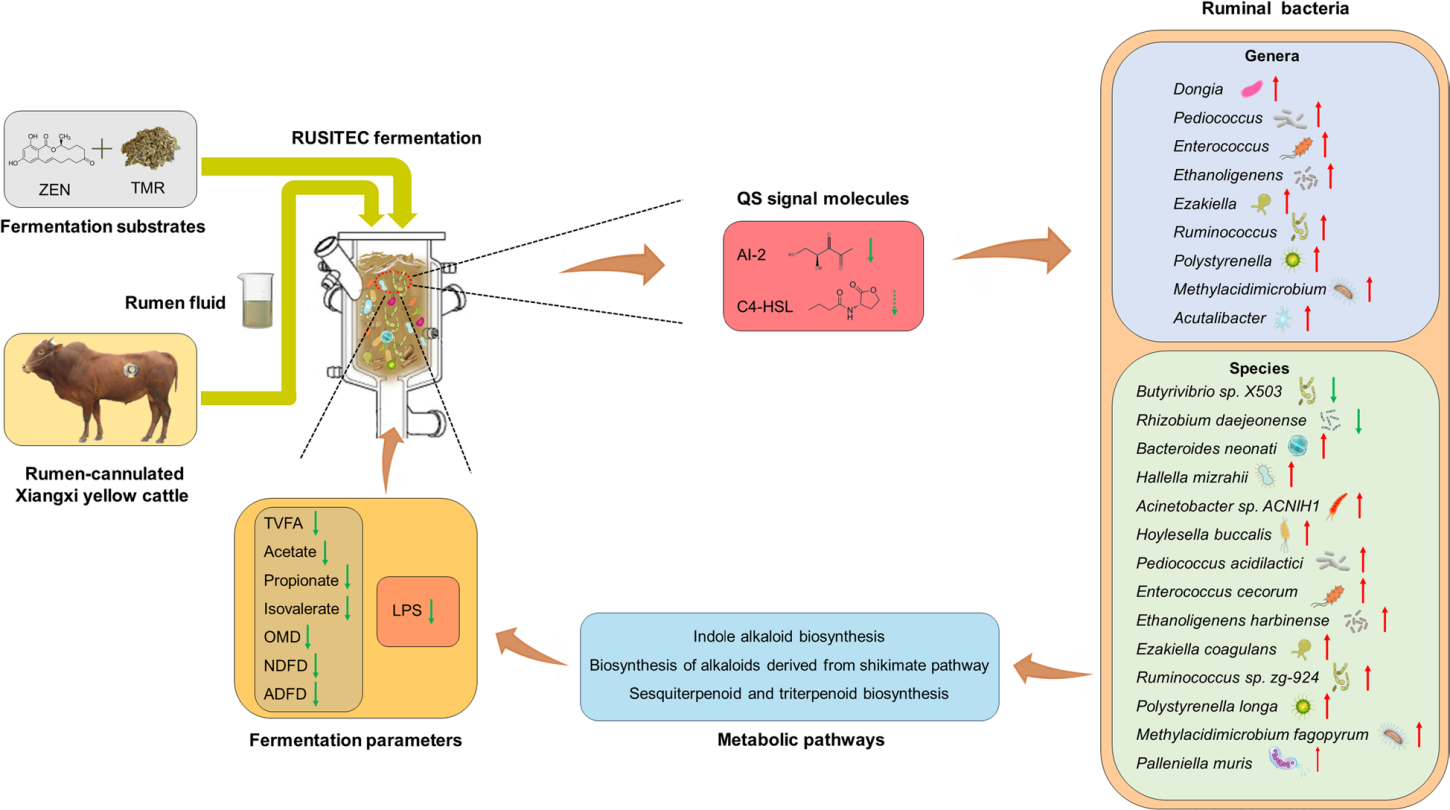
Overview summary of the present study. The green dotted down arrow represents tendentious (*P* < 0.1) decrement. The red up arrow represents significant (*P* < 0.05) increment. The green down arrow represents significant (*P* < 0.05) decrement

To the best of currently available knowledge, the current study provided the first proof demonstrating the potential roles of QS in the reactions of ruminal microbes to the ZEN exposure in a RUSITEC system. This investigation revealed associations of the AHLs/AI-2 molecules with the fluctuations of ruminal bacterial populations and rumen metabolisms by ZEN contamination, which could to a certain extent account for the deterioration of rumen fermentation (Fig. 7).

## Conclusion

In the RUSITEC system of the present trial, the ZEN contamination negatively affected the AHLs/AI-2 QS signals, which was demonstrated to be associated with the variations in specific rumen fermentation parameters, ruminal bacterial taxa, and ruminal metabolic pathways. These interconnections suggested the possible participation of QS in the responses of rumen microbiota to ZEN challenge, and might to a certain extent contribute to the inhibition of rumen fermentation. This study gained novel insights into the QS mediation in the influences of ZEN on the ruminal microflora, and deeper explorations were necessitated to decipher and testify the mechanisms of these correlations noted in the present study.

## Supplementary Information

Additional file 1: Fig. S1. Effects of ZEN on the ruminal metabolomic profiles through pairwise analysis.

Additional file 2: Fig. S2. Effects of ZEN on the ruminal metabolic pathways through pairwise analysis.

Additional file 3: Table S1. Sequencing summary.

Additional file 4: Table S2. OTU and relevant taxonomy.

Additional file 5: Table S3. Annotated taxa at different levels across samples.

Additional file 6: Table S4. Differential metabolites between treatments.

Additional file 7: Table S5. Commonly differential metabolites across treatments.

Additional file 8: Table S6. Commonly differential metabolic pathways across treatments.

Additional file 9: Table S7. Differential metabolic pathways between treatments.

## Data Availability

All the raw sequences obtained during the sequencing in this study were available at the NCBI database with the accession number PRJNA1250112.

## Abbreviations

ADF: Acid detergent fiber
AHLs: Acyl-homoserine lactones
AI-2: Autoinducer-2
Ca: Calcium
CON: Control
CP: Crude Protein
DM: Dry matter
DPD: (S)-4,5-dihydroxy-2,3-pentanedione
EE: Ether extract
HPLC-FD: High performance liquid chromatography equipped with a fluorescence detector
HSL: Homoserine lactone
KEGG: Kyoto Encyclopedia of Genes and Genomes
LDA: Linear discriminant analysis
LEfSe: Linear discriminant analysis effect size
LPS: Lipopolysaccharide
MCP: Microbial protein
MIMOSA2: Model-based integration of metabolite observations and species abundances 2
NDF: Neutral detergent fiber
NH_3_-N: Ammonia nitrogen
OTU: Operational taxonomic unit
P: Phosphorus
PCA: Principal components analysis
PCoA: Principal coordinate analysis
PICRUSt2: Phylogenetic investigation of communities by reconstruction of unobserved states 2
PLS-DA: Partial least squares discriminant analysis
QQ: Quorum quenching
QS: Quorum sensing
RUSITEC: Rumen simulation technique
SRA: Sequence read archive
TMR: Total mixed ration
TVFA: Total volatile fatty acid
UHPLC-MS: Ultra-high performance liquid chromatography-mass spectrometry
VFAs: Volatile fatty acids
VIP: Variable importance in projection
ZEN: Zearalenone
